# The Impact of Cancer Stem Cells in Colorectal Cancer

**DOI:** 10.3390/ijms25084140

**Published:** 2024-04-09

**Authors:** Petru Radu, Mihai Zurzu, Anca Tigora, Vlad Paic, Mircea Bratucu, Dragos Garofil, Valeriu Surlin, Alexandru Claudiu Munteanu, Ionut Simion Coman, Florian Popa, Victor Strambu, Sandu Ramboiu

**Affiliations:** 1 Tenth Department of Surgery, University of Medicine and Pharmacy “Carol Davila” Bucharest, 050474 Bucharest, Romania; petru.radu@umfcd.ro (P.R.); anca.tigora@drd.umfcd.ro (A.T.); vlad.paic@drd.umfcd.ro (V.P.); mircea.bratucu@umfcd.ro (M.B.); dragos.garofil@umfcd.ro (D.G.); ionut.coman@umfcd.ro (I.S.C.); florian.popa@spcaroldavila.ro (F.P.); victor.strambu@umfcd.ro (V.S.); 2 Sixth Department of Surgery, University of Medicine and Pharmacy of Craiova, Craiova Emergency Clinical 7 Hospital, 200642 Craiova, Romania; valeriu.surlin@umfcv.ro (V.S.); alexandru.munteanu@umfcv.ro (A.C.M.); sandu.ramboiu@umfcv.ro (S.R.); 3 General Surgery Department, “Bagdasar-Arseni” Clinical Emergency Hospital, 12 Berceni Road, 041915 Bucharest, Romania

**Keywords:** colorectal cancer, cancer stem cells, colorectal cancer stem cells, signaling pathways

## Abstract

Despite incessant research, colorectal cancer (CRC) is still one of the most common causes of fatality in both men and women worldwide. Over time, advancements in medical treatments have notably enhanced the survival rates of patients with colorectal cancer. Managing metastatic CRC involves a complex tradeoff between the potential benefits and adverse effects of treatment, considering factors like disease progression, treatment toxicity, drug resistance, and the overall impact on the patient’s quality of life. An increasing body of evidence highlights the significance of the cancer stem cell (CSC) concept, proposing that CSCs occupy a central role in triggering cancer. CSCs have been a focal point of extensive research in a variety of cancer types, including CRC. Colorectal cancer stem cells (CCSCs) play a crucial role in tumor initiation, metastasis, and therapy resistance, making them potential treatment targets. Various methods exist for isolating CCSCs, and understanding the mechanisms of drug resistance associated with them is crucial. This paper offers an overview of the current body of research pertaining to the comprehension of CSCs in colorectal cancer.

## 1. Introduction

CRC ranks as the third leading cause of cancer-related fatalities on a global scale [1]. Rather than being a singular consistent ailment, accumulating evidence suggests that CRC represents a spectrum of diseases characterized by diverse molecular profiles [2,3,4]. This inherent diversity presents formidable challenges in the pursuit of precision medicine via molecular-targeted therapies. Recent advancements in comprehensive molecular profiling and pathology examinations of CRC have substantially enriched our understanding of the genomic and epigenomic landscapes of these malignancies [4]. Consequently, CRC can now be stratified into biologically and clinically meaningful subtypes. This heightened comprehension of CRC’s molecular pathology has metamorphosed CRC diseases from a formerly enigmatic and heterogeneous group of conditions with variable clinical courses into distinct molecular categories [4]. This transformation paves the way for the implementation of personalized therapeutic approaches and enhanced patient care within the realm of CRC [5,6,7,8].

While European nations witness a declining trend in CRC incidence and mortality rates, the opposite trajectory unfolds in rapidly transitioning regions, notably in parts of Africa and South Asia [9]. The TNM staging system assumes a pivotal role in categorizing patients based on disease stage, employing anatomical criteria [10]. Beyond its prognostic utility, it serves as a compass for treatment decisions, taking into account overall health, tumor mutation status, and mismatch repair (MMR) status [11,12]. The arsenal of treatment options for CRC encompasses surgical resection, systemic therapies encompassing chemotherapy, targeted therapeutics, and immunotherapy, in addition to localized and palliative interventions [13,14,15].

Based on the latest data and study results, it is projected that the mortality rates associated with rectal and colon cancer will see a significant upswing by the year 2035. The current findings indicate an anticipated increase of approximately 60% in the mortality rate for rectal cancer, while colon cancer is expected to experience an even more substantial rise, with a projected increase of around 71.5% [16,17]. These estimates may vary by region and are often associated with factors such as economic development, highlighting the potential role of colorectal cancer as an indicator of a nation’s socioeconomic progress [17]. Individual lifestyle choices, including considerations such as body weight and dietary preferences, wield substantial influence over the observed surge in disease incidence [18]. Consistent followup examinations and preventive measures, including the maintenance of a well-balanced diet, constitute vital components of secondary prevention strategies.

Regrettably, treatment failures are not uncommon, frequently ascribed to the emergence of multidrug resistance (MDR) during or after therapy. Additionally, drug resistance can precipitate relapse, a phenomenon termed minimal residual disease (MRD) [9,19]. Both MDR and MRD can be traced back to a specific subset of tumor cells known as CCSCs, endowed with the capacity for self-renewal and differentiation into multiple lineages [19]. These CCSCs play a pivotal role in the initiation of tumors, their dissemination, resistance to therapeutic interventions, and the development of metastases. To further complicate treatment, the tumor microenvironment (TME) and metabolic adaptability exert selective pressure on cancer cells, fostering chemoresistance and propelling cancer progression. Consequently, the development of innovative therapies targeting CCSCs, while duly considering the TME and tumor metabolism, emerges as a promising strategy in the battle against therapy resistance [20,21].

## 2. Cancer Stem Cells

Looking back to the mid-19th century, German pathologist Rudolf Virchow emerges as the trailblazer of the CSC hypothesis, dating his pioneering work as far back as 1855 [22]. Virchow’s groundbreaking research laid the cornerstone for the hypothesis, suggesting that dormant embryonic-like cancer cells within mature tissues serve as a driving force behind cancer development. A significant breakthrough arrived in 1994 when Lapidot provided compelling empirical evidence that substantiated the CSC hypothesis; cells were isolated based on the expression of surface markers such as CD34+ and CD38−. Lapidot’s groundbreaking work involved the isolation of these cells, primarily identified through the expression of specific surface markers like CD34+ and CD38−. This milestone was achieved by effectively inducing leukemia in immunocompromised mice through the transplantation of human cells [23].

In subsequent research endeavors, CSCs have been identified in various tumor types, including both solid and nonsolid malignancies, underscoring their significance within the tumor microenvironment. These CSCs exhibit remarkable diversity, exemplified by the expression of unique surface markers (CD133, CD44, etc.), highlighting their heterogeneity across different cancer categories [24]. Importantly, CSCs possess the remarkable ability to instigate tumor formation through mechanisms involving self-renewal and differentiation into various cellular subtypes, thus adding to the complexity of cancer biology. The intricate regulation of CSC activities involves a multitude of factors, both intracellular and extracellular, many of which hold promising potential as targets for innovative anticancer therapies [24,25,26,27].

### Colorectal Cancer Stem Cells

The question of where CSCs in CRC originate from has generated significant debate, and multiple hypotheses have been advanced to explain their emergence. One prevailing notion is that CCSCs are intricately linked with the acquisition of malignant molecular and cellular alterations [28,29]. These alterations may arise through two principal mechanisms: first, the gradual accumulation of genetic and epigenetic changes within a subset of stem/progenitor cells and ordinary tumor cells, leading them down a malignant path. Second, it is posited that somatic cells can undergo a process of dedifferentiation, driven by a complex interplay of genetic mutations and environmental influences [30,31]. This dedifferentiation process may ultimately contribute to the emergence of CCSCs. This ongoing debate underscores the intricate nature of CSC origins and highlights the need for further research to elucidate the precise mechanisms involved. CCSCs manifest distinct characteristics closely associated with tumorigenesis and therapy resistance, contributing significantly to disease initiation, progression, and recurrence [32]. This unique subset of cells forms a reservoir of drug-resistant elements within the tumor, often responsible for post-chemotherapy relapses referred to as minimal residual disease (MRD) and the establishment of distant metastases. Consequently, CCSCs emerge as pivotal players in the intricate landscape of colorectal cancer, driving its relentless expansion and posing substantial therapeutic challenges [32,33].

Notably, CCSCs possess the exceptional ability to generate heterogeneous tumors that can be serially transplanted into immunocompromised mice, faithfully replicating the primary tumor’s characteristics [34]. Furthermore, their prolific nature endows CCSCs with the capacity to give rise to disseminated metastatic tumors, further complicating the clinical management of colorectal cancer and necessitating innovative therapeutic approaches [31]. Nevertheless, investigating CCSCs presents substantial challenges, chiefly due to their limited prevalence within the tumor microenvironment. Moreover, the intricate spectrum of phenotypic and functional variations among CCSCs further complicates the task of identifying and isolating them [35]. As a result, while the prospect of therapies designed to target CCSCs offers considerable promise in securing long-lasting clinical outcomes, achieving this goal depends significantly on the advancement of resilient technologies proficient in efficiently detecting and capturing these enigmatic cellular components [35,36,37,38].

## 3. Isolation and Identification of CCSCs

A wide array of methodologies and strategies are employed in the isolation of CCSCs. These methods encompass a spectrum of techniques and tools aimed at identifying and isolating this unique subset of cancer cells with precision and specificity. The selection and application of these approaches are driven by the need to capture the distinctive properties and behaviors of CCSCs, a task that has garnered considerable attention in cancer research [33,39].

### 3.1. Utilizing Surface Markers

Numerous stem cell markers have been investigated in the context of CCSCs, reflecting their diverse characteristics and behaviors. However, comprehending and isolating CCSCs prove to be intricate tasks given their multifaceted and ever-evolving nature. Within the realm of colorectal CSC identification, a spectrum of surface markers have emerged as pivotal players. Notably, CD44, CD133, CD166, Lgr5, ALDH1, and EpCAM have taken the spotlight as primary markers closely associated with CCSCs. These markers not only serve as identifiers but also wield significant biological functions that contribute to the unique attributes of CCSCs. Moreover, a broader spectrum of universally recognized CSC markers come into play, each extending beyond their surface marker roles to encompass complex and multifaceted biological functionalities. These include Nanog, Sox2, Oct-4, CD51, CD24, CD26, and CD29, among others, painting a comprehensive picture of the intricate landscape of CCSC markers and their diverse roles in colorectal cancer (Table 1) [9,35,40].

In conclusion, a multitude of markers play a pivotal role in the identification and isolation of CCSCs, each possessing distinct capabilities and characteristics. Among these markers, CD133, Lgr5, Bmi-1, CD26, and CD44v6 stand out as independent identifiers of CCSCs, capable of marking and isolating these stem cells individually. Their unique properties provide valuable options for researchers seeking to study and target CCSCs. Furthermore, in addition to these standalone markers, there are others in the repertoire that effectively identify CCSCs. Nevertheless, in many cases, these markers are employed in combination with one or more of the previously mentioned markers. These combinations offer a more refined and comprehensive approach to CCSC isolation, enriching our understanding of these critical cell populations. These markers collectively contribute to the advancement of our knowledge regarding CCSC biology and their role in colorectal cancer. They serve as indispensable tools for researchers, offering versatility in the isolation and investigation of CCSCs using techniques like magnetic-activated cell sorting (MACS) and fluorescence-activated cell sorting (FACS). The combined utilization of these markers empowers researchers to delve deeper into the intricacies of CCSCs, ultimately shedding light on their significance in colorectal cancer progression and treatment resistance [40].

### 3.2. Isolation within In Vitro Cultures

Stem cells, whether normal or neoplastic, are characterized by their unique ability to self-renew and give rise to differentiated cells over the long term. In the context of colorectal CCSCs, in vitro culture methods play a crucial role in assessing these fundamental properties. These methodologies also offer a means to isolate CCSCs directly from patient tissues without prior marker selection. Culturing intestinal CCSCs presents challenges similar to those encountered in other normal and tumor stem cell cultures, particularly the preservation of self-renewal capacity. Progress in this field has been achieved through the identification of components for defined culture media and an enhanced understanding of the molecular and cellular mechanisms governing stemness. One key distinction between solid tumor stem cell cultures and leukemia cultures is the requirement for adhesion in the former. This adhesion requirement is addressed through two prevalent methodologies: spheroid and organoid cultures [41].

Spheroid Cultures: These are grown in low-adherence cell culture conditions, where dissociated cells from patient tissues generate self-adhering floating clusters or spheroids. Spheroid cultures enable substantial expansion and maintenance of xenograft-initiating capability, making them ideal for high-throughput molecular analyses and drug testing. They have been instrumental in identifying potential CCSC-targeted agents [42,43,44,45]. 

Organoid Cultures: In this approach, cells are embedded in a basement membrane matrix, often Matrigel, to fulfill the adhesion requirement [46]. Organoids more faithfully reproduce the original tumor’s architecture, forming complex structures. While they offer architectural fidelity, organoid cultures can be more resource-intensive due to Matrigel use and the challenge of releasing cells from the embedding matrix. Patient-derived organoids have proven valuable in predicting patients’ responses to drugs and radiation [47,48].

Cultures of spheroids and organoids incorporating human cCSCs are amenable to genetic alterations, providing a platform for investigating the function of established or potential oncogenic markers. Highlighting a particularly notable set of experiments, the integration of organoid cultures with CRISPR technology facilitates examination of the genetic alterations driving cancer development. Specifically, the stepwise introduction of mutations into genes such as APC, SMAD4, TP53, and KRAS enables organoids to mimic adenoma-to-carcinoma progression, revealing a successive decline in cell dependence on niche signals [49,50]. Recent investigations have also aimed to map the genetic changes associated with the capacity for metastasis. Crucially, cCSCs maintained in either spheroid or organoid formats are viable for creating xenografts in immunocompromised rodents [51,52,53]. The transplantation of in vitro cultured or genetically tailored intestinal CSCs represents a critical strategy for elucidating cCSC dynamics.

### 3.3. CCSC Isolation via Biophysical Characteristics

Sedimentation field-flow fractionation (SdFFF) is an innovative method that enables the gentle and label-free separation of CCSCs based on their biophysical characteristics, such as size, density, shape, and rigidity [54]. This technique has gained popularity in various fields, including oncology and stem cell research. Researchers have successfully used SdFFF to isolate distinct CCSC subpopulations from different human colorectal cancer cell lines [34,54]. These enriched CCSC fractions have been further studied using models like the chick embryo chorioallantoic membrane (CAM) to explore carcinogenesis and treatment sensitivity. One of the key advantages of SdFFF is that it eliminates the need for cell labeling, but it does require a substantial number of cells and is time-consuming [55].

SdFFF emerges as a cutting-edge and promising technique for the isolation and segregation of CCSCs, capitalizing on their distinctive biophysical attributes. In contrast to conventional methods that frequently necessitate cell labeling or reliance on specific markers, SdFFF offers an innovative approach, ushering in fresh possibilities for CCSC exploration and potential clinical implications. This method hinges on the unique physical characteristics of CCSCs, encompassing factors like size, configuration, stiffness, and density, to facilitate their differentiation. It functions by exposing different cell subgroups to both the parabolic flow pattern generated by the mobile phase within the channel and a multifaceted external gravitational field produced by channel rotation [55].

## 4. CCSC Signaling Pathways

Stem cells are characterized by their ability to undergo self-renewal, orchestrated proliferation, differentiation into diverse cell types, and their responsiveness to specific signaling cascades. However, the abrupt disruption of these fundamental attributes, accompanied by the irregular activation of inactive oncogenes, can precipitate the emergence of CCSCs, thereby driving tumorigenesis. Several pivotal signaling pathways play a pivotal role in sustaining the stem cell-like traits of CCSCs, encompassing Hedgehog (Hh), Wnt/beta-catenin, Notch, and Hippo. These pathways consistently experience dysregulation within CCSCs and are imperative for upholding their stem cell-like characteristics. The Hh, Notch, and Wnt/beta-catenin signaling pathways wield substantial influence over the regulation of tumorigenesis in CCSCs. Consequently, therapeutic interventions designed to modulate these signaling pathways hold significant potential as innovative approaches for cancer therapy [56].

### 4.1. Wnt Signaling Pathway

The Wnt/β-catenin signaling pathway stands at the forefront of crucial biological processes, orchestrating embryonic development, stem cell maintenance, cellular renewal, and tissue homeostasis. This pathway’s functioning hinges on the β-catenin protein, a versatile molecule that serves as a transcription factor within the nucleus and a critical component of the cytoskeleton, underscoring its indispensable role in cellular growth and development. In the absence of Wnt ligands—specific hydrophobic secretory proteins—β-catenin is kept at bay through a finely tuned degradation process overseen by the β-catenin destruction complex, comprising Axin, GSK3, CK1, APC, and β-TrCP2. This regulatory mechanism ensures that β-catenin does not erroneously activate gene transcription by maintaining its levels in a tightly controlled manner [57,58,59,60]. 

The activation saga of the Wnt/β-catenin pathway unfolds when Wnt ligands bind to Frizzled receptors and LRP5/6 co-receptors on a cell’s surface, catalyzing a series of events that lead to the stabilization and nuclear translocation of β-catenin. Once in the nucleus, β-catenin collaborates with TCF/LEF transcription factors to kickstart the transcription of genes that are quintessential for pivotal biological functions, highlighting a meticulously orchestrated regulatory mechanism that is vital for cellular fate determination and proliferation. This pathway’s regulation is an exemplar of biological complexity, employing a multitude of antagonistic proteins such as WIF, DKK1, and sFRPs, which serve to inhibit pathway activation by directly binding to Wnt ligands or their receptors. Simultaneously, feedback mechanisms involving proteins like ZNRF3 and RNF43 modulate the pathway’s activity by downregulating Frizzled receptors. Conversely, the activity of R-Spondin exemplifies the nuanced regulation within this pathway, enhancing Wnt signaling by preventing the degradation of Frizzled receptors, thereby maintaining a critical balance in cellular signaling mechanisms [57,58,59,60].

**Figure 1:**
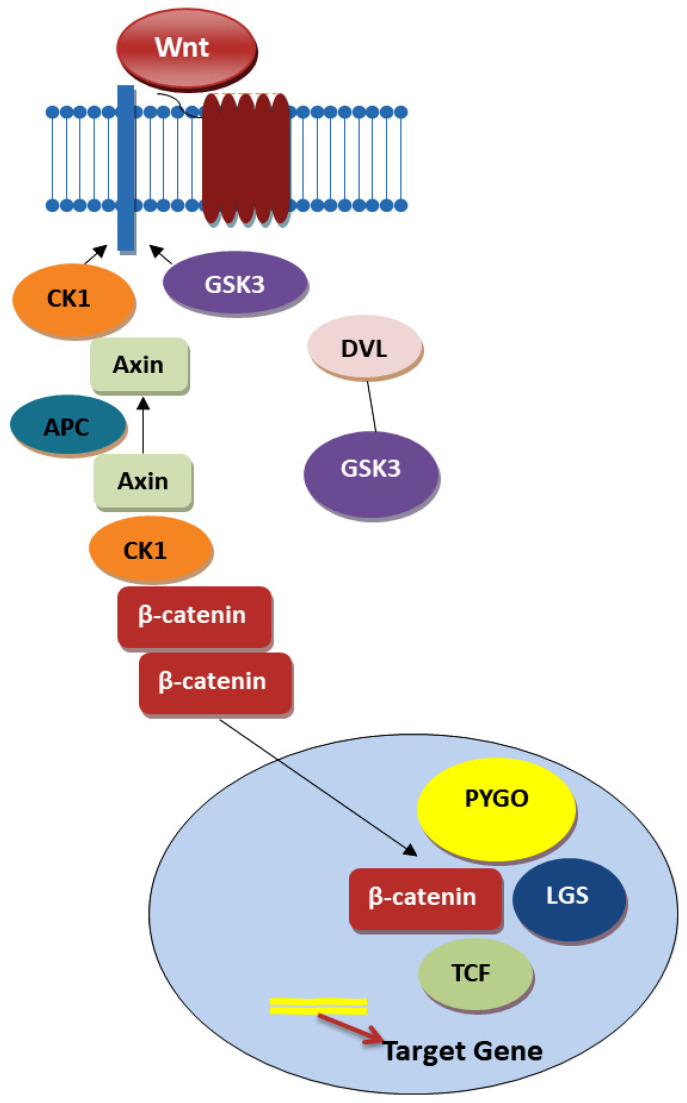
Canonical Wnt pathway—based on Refs. [61,62].

**Figure 2:**
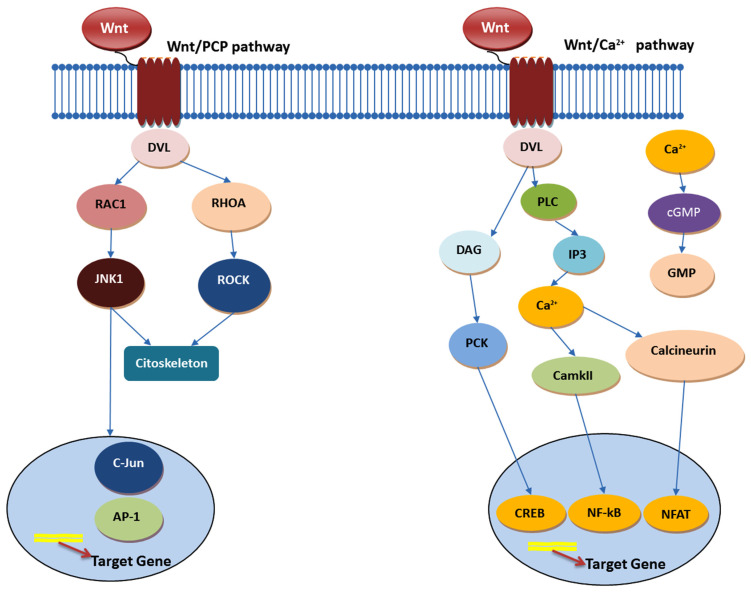
Noncanonical Wnt pathway—based on Refs. [61,62].

The role of TCF/LEF transcription factors within this pathway introduces an additional layer of regulation and specificity, with these factors exhibiting diverse functionalities based on their interaction with β-catenin. This complexity ensures that the pathway’s outcomes are finely tuned to the cellular context, allowing for a wide range of cellular outcomes based on the type of cell, its location, and its physiological state. In the realm of cancer, particularly epithelial cancers such as colon cancer, the Wnt signaling pathway plays a pivotal role in regulating stem cell self-renewal and differentiation. The canonical (Figure 1) and noncanonical pathways (Figure 2), triggered by various Wnt ligands, lead to distinct cellular outcomes: the former determining cell fate and the latter overseeing tissue polarity and cell movement. CCSCs, marked by high Wnt signaling activity, localize within specific niches, suggesting the potential of targeting the Wnt pathway as a therapeutic strategy to modulate the stem cell population [57,58,59].

The canonical pathway, especially, has been identified as a therapeutic target due to its role in the stabilization and accumulation of β-catenin, which, when unregulated, contributes to stem cell fate determination, cell proliferation, and the regulation of other pathways. Aberrations such as truncating mutations in APC lead to the pathological stabilization of β-catenin, thereby offering potential intervention points, such as TNIK inhibition, which has shown promise in blocking Wnt signaling and targeting CCSCs for eradication. Furthermore, the R-Spondin pathway, an activator of WNT signaling, emerges as a potential target, especially in cancers where RSPO translocations occur, offering avenues to inhibit cancer stem cell survival and proliferation [57,58,59,60].

### 4.2. Notch Signaling Pathway

**Figure 3:**
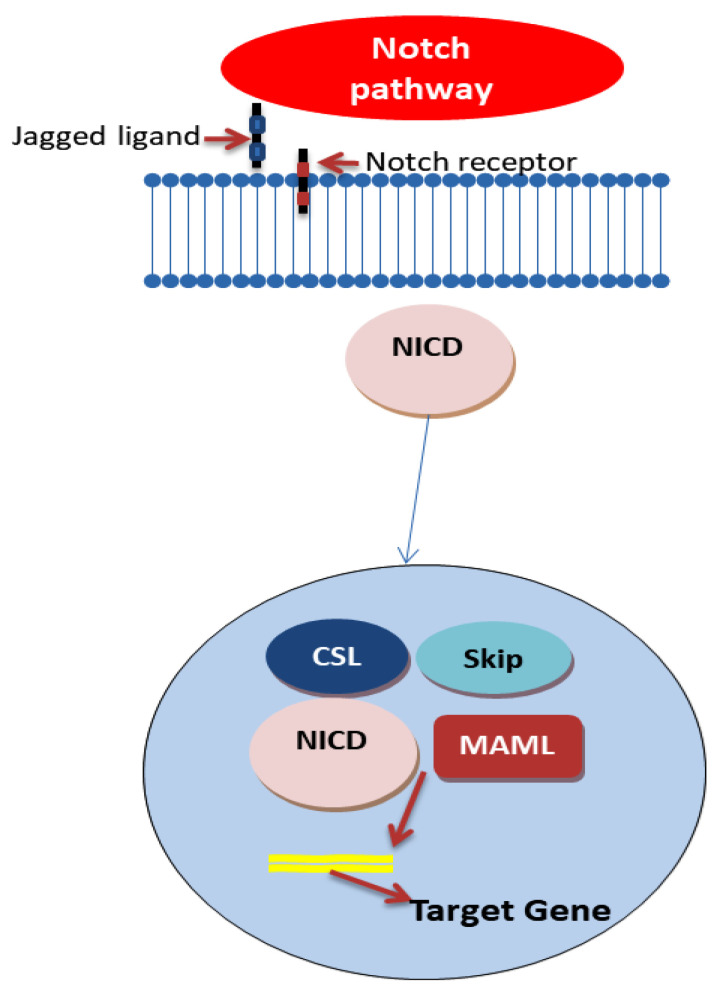
Notch pathway—based on Refs. [70,71].

The Notch signaling pathway (Figure 3) is crucial in regulating cell fate decisions, sustaining stem cell phenotypes, and contributing to tumor heterogeneity, particularly noted in CRC. This pathway’s dysregulation, often due to mutations that lead to its constitutive activation, is associated with the progression of CRC, resistance to chemotherapy, and impacts regarding tumor heterogeneity [63,64,65].

Notch signaling is recognized as a principal regulator of stemness and self-renewal in a wide array of solid cancers, including CRC. The pathway’s ligand/receptor specificity further influences tumor heterogeneity. For instance, Notch1/DLL1 signaling differentiation between “non-neuroendocrine” and “neuroendocrine” cell types in small cell lung cancer (SCLC) highlights Notch’s role in modulating transitions between CSCs with stem-like features and more differentiated tumor cells, suggesting that a similar mechanism might be at play in CRC. In the intestinal epithelium, Notch signaling maintains a delicate balance between stem cell preservation and differentiation. Aberrant activation of Notch receptors in CRC points to their role in bolstering the stemness of CRC cells, complicating treatment resistance and disease recurrence [66]. Notch signaling components, particularly the HES gene family, are markedly upregulated in CSCs, fostering cell survival and impeding differentiation [63,64,65,67].

The complexity of the Notch pathway is further exemplified by its role in CSC heterogeneity, facilitated through the asymmetric division regulated by Notch1 signaling. This process, influenced by epigenetic modifications and microenvironmental factors, maintains a balance between CSC states. The modulation of Notch signaling by various elements, including miRNA and chromatin methylation, underscores the sophisticated regulatory mechanisms that govern its impact on CSC dynamics. Given the pathway’s implication in CRC cell stemness, particularly through JAG1/Notch1 interactions, targeting Notch signaling presents an appealing yet challenging strategy for CRC treatment. The pathway’s influence extends to apoptosis inhibition, affecting tumor cell differentiation, proliferation, and maintaining CSC pluripotency. The pursuit of selective Notch pathway modulators, capable of targeting cancerous cells without harming healthy tissue, is critical, emphasizing the need for an in-depth understanding of this signaling pathway’s role in CRC and the broader context of solid tumors [63,64,65,67]. 

The current therapeutic approaches targeting the Notch pathway, including gamma-secretase inhibitors (GSI) and ADAM17 inhibitors, demonstrate promise in reducing colorectal CSCs’ self-renewal and encouraging their differentiation. Specifically, targeting Notch ligands, like DLL4, with neutralizing antibodies has also shown therapeutic potential, offering insights into strategies that could disrupt CSC maintenance while mitigating broader pathway inhibition’s side effects [63,64,65]. Furthermore, a study conducted by Anqi Lin et al. regarding Notch has shown that mutations in the Notch signaling pathway significantly influence the efficacy of immune checkpoint inhibitors (ICIs) in colorectal cancer (CRC) treatment. Patients with Notch mutations (NOTCH-MT) exhibited notably longer overall survival when treated with ICIs compared to those without mutations (NOTCH-WT). This survival benefit was not observed in CRC patients who did not undergo ICI therapy. The research also highlighted an enriched immune microenvironment in NOTCH-MT tumors, suggesting a more robust response to ICIs due to increased immunogenicity. Gene enrichment analysis further revealed that NOTCH-MT tumors exhibited upregulation in immune activation pathways and downregulation in pathways like Wnt signaling and fatty acid metabolism [68]. In parallel, the work of Francesca Negri et al. further supports the significance of Notch signaling in CRC outcomes, focusing on the expression of NICD and Jag1. Their study, involving 111 patients, found that high NICD and Jag1 expression correlates with shorter progression-free survival and poor treatment response. Notably, NICD emerged as an independent predictor of survival. The research also leveraged a multiomic approach, enhancing the ability to predict patient outcomes by identifying radiomic features indicative of survival length [69]. 

### 4.3. Hedgehog Signaling Pathway

**Figure 4:**
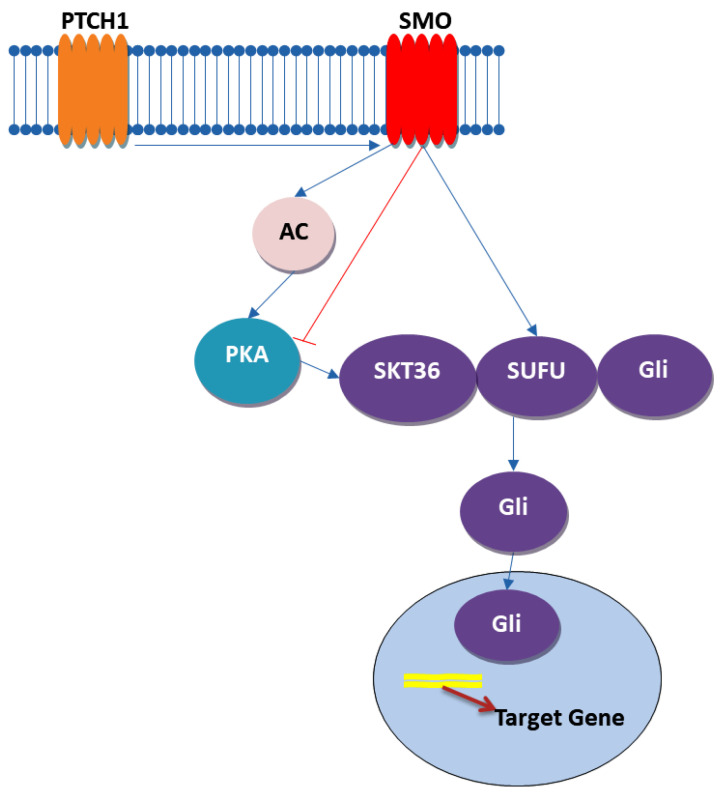
Hh pathway—based on Refs. [76,77].

The Hedgehog (Hh) signaling pathway (Figure 4), a crucial regulator of organ development and patterning in both vertebrates and invertebrates, plays a pivotal role in colorectal cancer (CRC) as well. It is fascinating how a pathway named after the spiky appearance of *Drosophila larvae* has evolved into a cornerstone of developmental biology and cancer research. The pathway operates through the production and secretion of Hh ligands like Indian Hedgehog (IHH), Sonic Hedgehog (SHH), and Desert Hedgehog (DHH), which undergo significant post-translational modifications to enable their signaling capabilities. These modifications include palmitoylation by Hh acetyltransferase (HHAT) and autocatalytic cleavage, attaching lipid moieties that anchor them for cellular communication [72,73]. Dispatched1 (DISP1) and its cofactor SCUBE2 play instrumental roles in the secretion and dissemination of Hh ligands, while Heparan sulfate proteoglycans (HSPGs) facilitate their transport through tissue. These mechanisms underscore a sophisticated system of long-range signal transmission that is vital for tissue patterning and organ development. At the receiving end, transmembrane proteins Patched 1 (PTCH1) and Smoothened (SMO) are the primary receptors and signal transducers. In the absence of Hh ligands, PTCH1 inhibits SMO, preventing signal propagation. However, upon ligand binding, this inhibition is lifted, allowing SMO to initiate downstream signaling that leads to the activation of Glioma-associated oncogene (GLI) transcription factors. These factors then modulate the expression of target genes, including those involved in cell differentiation, proliferation, and survival [72,73].

Abnormal activation of the Hh pathway can contribute to the onset, expansion, and persistence of cancer, especially in aggressive and drug-resistant tumors characterized by an excessive presence of Hh signaling components. Scientific investigations have unveiled the involvement of the Hedgehog-GLI (HH-GLI) pathway in preserving the ability of CCSCs and CD133+ colon CSCs to self-renew. When certain ligands, like the Shh exo-secretion ligand, bind to transmembrane receptor PATCHED1 (PTCH1), they initiate the HH-GLI pathway. This initiation triggers a series of interconnected events culminating in the activation of the Gli2 transcription factor. This activated Gli2 has a profound impact on cell proliferation, regulatory mechanisms, and fate determination, influencing the expression of specific genes. Inhibition of the HH-GLI pathway, either directly or indirectly, holds the potential to eliminate both bulk tumor cells and the CSC population within tumors. Compounds like cyclopamine and GDC-0449 have shown effectiveness in targeting this pathway and reducing stem cell markers in cancer cells. Combining Hh inhibitors with conventional chemotherapeutic agents and radiation therapy may provide a promising approach to prevent tumor relapse and enhance patient outcomes [73,74,75].

However, optimizing the use of Hh inhibitors in combination with standard therapies, as well as tailoring them to specific cancer subtypes based on Hh pathway activation, require further investigation. A deeper understanding regarding how Hh signaling contributes to CSC maintenance and its clinical integration with conventional treatments are essential to fully realize the therapeutic potential of this strategy.

### 4.4. Hippo Signaling Pathway

**Figure 5:**
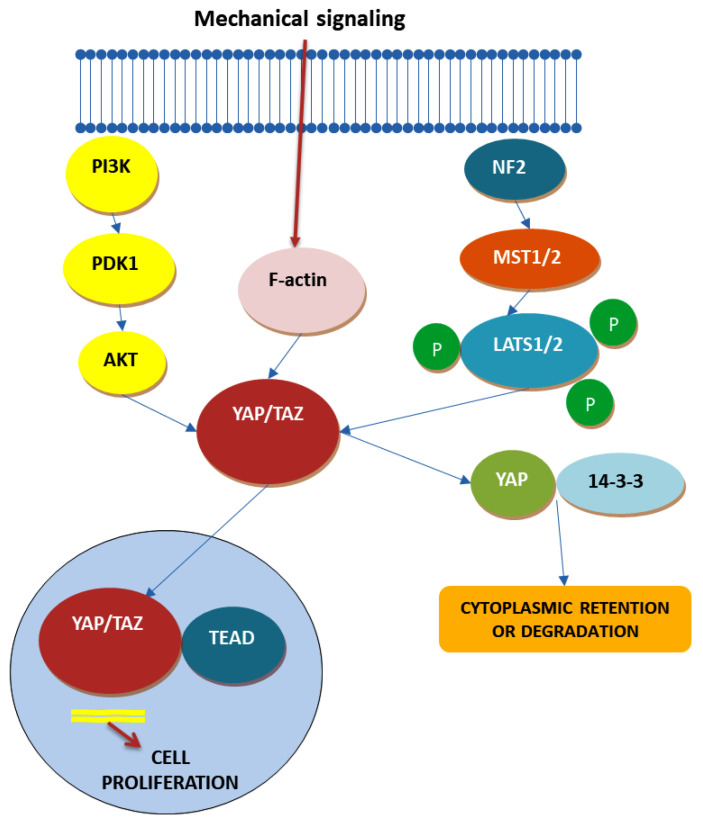
Hippo pathway—based on Refs. [83,84,85].

More than a decade ago, a groundbreaking discovery unveiled one of the most influential pathways governing the characteristics of cancer stem cells—known as the Hippo pathway (Figure 5). This intricate pathway not only exerts control over tissue development and regeneration and tumorigenesis but also, as emerging research reveals, wields significant sway over the realm of cancer stem cell biology [75]. This domain encompasses processes like epithelial–mesenchymal transition (EMT), resistance to drugs, and the ability to self-renew [75]. The origins of this revelation can be traced back to insights derived from *Drosophila melanogaster* and subsequently validated in transgenic mouse models [75]. The Hippo pathway comprises two major constituents: the cytoplasmic kinase module and the nuclear transcription module. Within the oncogenic transcriptional module, key actors such as yes-associated protein (YAP) and transcriptional co-activator with PDZ-binding motif (TAZ) take center stage. Together with the TEA domain family member (TEAD), YAP/TAZ function as transcriptional coactivators, orchestrating critical gene expression [75].

The activation of the Hippo pathway plays a pivotal role in regulating cell growth inhibition through cell–cell contact. Under normal physiological conditions, observed in processes like wound healing and embryonic development, the Hippo pathway springs into action upon the release of specific proteins during cell–cell contact. However, when cell–cell contact inhibition diminishes, as observed in tumorigenesis during EMT, YAP/TAZ become hyperactive, giving rise to tumorigenesis. Beyond cell contact, YAP/TAZ are also responsive to mechanical cues, including factors such as extracellular matrix stiffness, cell adhesion, cell geometry, and cytoskeleton tension. Moreover, extracellular nutrients and environmental stressors wield regulatory control over the Hippo pathway [66,75,78].

Within the context of CRC, numerous studies have underscored the involvement of YAP. Elevated YAP expression has been associated with higher histological grades, enrichment of colon stem cell characteristics, metastatic proclivities, and cancer progression. Some investigations have even indicated resistance to cetuximab therapy due to YAP upregulation. On the flip side, certain conflicting studies have suggested that YAP may, in fact, function as a tumor suppressor gene, underscoring its context-dependent roles. Understanding YAP’s precise functions in CRC remains an ongoing challenge, but its role in maintaining stemness and tissue equilibrium cannot be overlooked. Its presence primarily at the crypt base, in contrast to its absence from villi, hints at YAP’s role in preserving the undifferentiated state of stem cells by binding to TEAD transcription factors. Recent studies have begun to unveil YAP’s dual role in regulating intestinal stem cells as hyperactivation expands these cells while its deletion impairs regeneration following experimental damage. Researchers have explored the potential of targeting YAP with small-molecule modulators in various cancer types. These modulators can be categorized into three main groups: those regulating upstream molecules of YAP and its downstream transcriptional activity, those modulating YAP phosphorylation and impeding its nuclear translocation, and those inhibiting YAP to disrupt its interaction with TEAD1. Oligomeric proanthocyanidins (OPCs), found in fruits and vegetables, have exhibited antitumorigenic and anti-CCSC properties by inhibiting YAP/TAZ and thus the Hippo pathway. Verteporfin, known for enhancing phototherapy in macular degeneration, has demonstrated antitumor effects unrelated to YAP inhibition in CRC. It has also shown promise in reversing paclitaxel resistance attributed to YAP overexpression in certain cells [66,79,80,81,82]. 

However, a significant challenge in developing YAP-targeting small molecules lies in its dual role as both an oncogene and a tumor suppressor. Deciphering whether stimulating or inhibiting YAP expression represents a more suitable strategy against CCSCs remains a topic that necessitates in-depth investigation and analysis.

## 5. CCSC and Tumor Microenvironment

In the landscape of modern oncology, the significance of TME has been increasingly acknowledged for its role in promoting cancer growth and the emergence of resistance mechanisms against pharmacological interventions, presenting significant challenges in the development of innovative cancer treatments. The behavior of tumor cells, including CSCs, is heavily influenced by the complex interplay of various cell types, blood vessels, lymph vessels, extracellular matrix (ECM), and signals from the surrounding microenvironment. This tumor niche orchestrates immune responses, induces the formation of cancer-associated fibroblasts (CAFs), mesenchymal stem cells (MSCs), endothelial cells (ECs), ECM remodeling, and secretion of soluble factors. The ECM, with its distinct composition, significantly impacts signaling pathways, cellular movements, invasion, and angiogenesis, while also serving as a barrier against chemotherapy and radiotherapy and contributing to hypoxia. Various growth factors and soluble agents present in the tumor microenvironment (TME), such as transforming growth factor-beta (TGF-β), interleukin-6 (IL-6), fibroblast growth factor (FGF), and hepatocyte growth factor (HGF), play crucial roles in tumor growth, therapy resistance, and the induction of stemness properties [86,87]. In 2010, Vermeulen and colleagues demonstrated that factors secreted by myofibroblasts, especially hepatocyte growth factor (HGF), amplify Wnt signaling in colon cancer cells. This action can revert more differentiated tumor cells back to a CSC phenotype, observed both in laboratory and live animal studies [88]. Several key cell types within the TME, including immune cells like tumor-associated macrophages (TAMs), natural killer (NK) cells, dendritic cells (DCs), T lymphocytes, B lymphocytes, and myeloid-derived suppressor cells (MDSCs), form intricate networks that can either support or counteract tumor progression. Inflammation and hypoxia, essential conditions for tumor growth, accelerate tumor initiation and proliferation by promoting genomic instability and activating key signaling pathways such as STAT3 and NF-κB. Chronic inflammation mediated by cytokines and growth factors contributes to activation of the transcription factors involved in cellular proliferation and stemness induction, linking CSCs to the inflammatory milieu. Interleukin-6 (IL-6), a prominent factor in inflammation, enhances CSC survival, invasion, and resistance properties. CSCs also establish their niche through interactions with the TME, mediated by metabolites, exosomes, cytokines, and growth factors, which regulate processes such as metastasis, angiogenesis, immune evasion, and drug resistance [89,90].

Metabolic plasticity in CSCs is intricately influenced by various factors within the tumor microenvironment (TME), including cancer-associated fibroblasts (CAFs), endothelial cells, inflammatory mediators, and hypoxia. These factors collectively shape the metabolic profile of CSCs, impacting their self-renewal capacity, therapeutic resistance, and stemness properties. Activation of signaling pathways such as NF-κB and PI3K/Akt by inflammatory cytokines within the TME plays a pivotal role in promoting CSC self-renewal. Inflammatory agents contribute to the activation of these pathways, thereby enhancing CSC survival and proliferation [91]. This inflammatory microenvironment fosters a niche that supports CSC maintenance and propagation. Hypoxia, a hallmark feature of solid tumors, exerts profound effects on CSC metabolism. In response to low oxygen levels, CSCs undergo metabolic reprogramming characterized by increased glycolysis, altered lipid metabolism, and enhanced autophagy. These metabolic changes not only enable CSCs to survive and proliferate under hypoxic conditions but also confer resistance to therapy, making them a challenging population to eradicate [91]. 

Endothelial cells, which form the inner lining of blood vessels, are essential for CSC maintenance and function. Through the secretion of key factors such as Notch and Wnt ligands, endothelial cells sustain CSC self-renewal and stemness properties. Additionally, endothelial cells facilitate immune modulation within the TME, influencing the behavior of CSCs and other immune cell populations. By promoting angiogenesis, endothelial cells contribute to the vascularization of tumors, thereby facilitating CSC survival and dissemination. Immunomodulation in the TME compromises immune cell function and promotes CSC survival and invasion. NK cells, T cells, and macrophages play pivotal roles in antitumor immunity, but their functions are hindered by TME-induced metabolic changes and immunosuppressive factors [92].

CAFs, the predominant stromal cells within the TME, play multifaceted roles in tumor progression. They remodel the extracellular matrix (ECM), promote angiogenesis, and actively modulate CSC metabolism by secreting growth factors and cytokines that fuel CSC migration, proliferation, and cytokine secretion. By creating a supportive metabolic niche, CAFs contribute to the maintenance and expansion of CSC populations within tumors. Additionally, CAFs promote CSC stemness, thereby contributing to therapy resistance [93]. CAFs play a pivotal role in the TME by promoting tumor growth and resistance to therapy. They modulate the ECM, making drug penetration difficult. Interestingly, RT paradoxically promotes CAF proliferation, leading to radioresistance [94]. Additionally, CAFs contribute to the creation of an immunosuppressive TME, aiding cancer cells in evading immune surveillance. Different subpopulations of CAFs influence immune suppression through various mechanisms, thereby contributing to therapy resistance. Furthermore, CAFs are involved in drug resistance mechanisms, obstructing drug delivery and promoting cell adhesion-mediated drug resistance. Hypoxia in the TME influences CAF recruitment and activity, contributing to tumor progression and therapy resistance [90].

Hypoxia-inducible factor 1 (HIF-1) plays a crucial role in CSC resistance to RT by regulating genes involved in stemness, angiogenesis, and tumor relapse. It protects tumor blood vessels and promotes cancer cell repopulation after RT. Moreover, angiogenesis in the TME affects the radiosensitivity of cancer cells. TME factors, including CAFs, influence angiogenesis, thereby impacting RT outcomes. Finally, HIF-1 regulates non-coding RNAs, including microRNAs and long non-coding RNAs, affecting cancer cell response to RT through various mechanisms [89].

## 6. CCSC and Drug Resistance

Recent studies have significantly advanced our understanding of CRC by shedding light on the intricate relationship between CCSCs and drug resistance mechanisms. Mateus de Almeida Rainho and colleagues conducted a study that delved deep into the role of CCSCs and mitochondria in chemotherapy resistance in CRC. Their research highlighted the crucial involvement of mitochondrial dynamics in sustaining chemoresistance through mechanisms such as mitophagy and metabolic shifts. By identifying adaptive strategies employed by CCSCs, including the upregulation of anti-apoptotic proteins and increased mitophagy, they underscored the importance of targeting mitochondrial pathways to counteract chemoresistance. Additionally, they advocated for the exploration of mitochondrial-targeted drugs (MTDs) as promising therapeutic avenues to impair the mitochondrial functions essential for CCSC survival [95].

Zhenzhen Wei et al. investigated resistance mechanisms to anti-EGFR therapy in CRC, particularly focusing on exosomes derived from multidrug-resistant (MDR) CRC cells. Their study demonstrated how MDR-derived exosomes convey resistance to cetuximab and enhance the stemness of CRC cells. By characterizing these exosomes and elucidating their role in mediating drug resistance, they provided valuable insights into potential therapeutic targets. Furthermore, their findings underscored the involvement of the PI3K/AKT signaling pathway and stem cell-associated genes in promoting cetuximab resistance, highlighting the complexity of CRC resistance mechanisms [96].

Yuchen Li and colleagues explored the efficacy of AT7867, an AKT inhibitor, against CSCs in CRC. Their study revealed significant inhibitory effects of AT7867 on CSC proliferation and stemness attributes, offering promising therapeutic potential. By elucidating the mechanism involving the downregulation of Ascl2 and interference with the Akt signaling pathway, they proposed a compelling strategy for targeting CSCs in CRC treatment [97]. 

Addressing chemoresistance in CRC, Yan Su’s study introduced diHEP-DPA as a promising therapy to counteract resistance to 5-fluorouracil (5-FU). Their research highlighted the synergistic effects of diHEP-DPA and 5-FU in reducing tumor size and weight, offering a hopeful avenue for enhancing CRC treatment efficacy. Mechanistic investigations revealed the ability of diHEP-DPA to mitigate 5-FU-induced activation of CSCs and suppress the infiltration of tumor-associated macrophages (TAMs), further emphasizing the complexity of CRC resistance mechanisms [98].

Mangiapane LR et al.’s study delved into the challenges of treating advanced CRC by focusing on therapy resistance mechanisms and the quest for more effective treatments. Their research highlighted tumor heterogeneity and the activation of survival pathways, particularly mutations in RAS and the epidermal growth factor receptor (EGFR), as key contributors to resistance against targeted therapies. They specifically investigated the role of CCSCs in tumor growth, spread, and resistance to chemotherapy. Their findings revealed that CCSCs exhibit high levels of CD44v6 and rely on the PI3K/AKT pathway for survival and proliferation. Moreover, they identified consistent expression of HER2 in CCSCs, suggesting a potential target for therapy. By targeting HER2 alongside PI3K and MEK, they proposed a triple-combination therapy to overcome therapy resistance in CRC [99].

Souvick Roy and colleagues explored a novel therapeutic strategy targeting CSCs in CRC using metformin and ICG-001. Their study aimed to disrupt the Wnt/β-catenin signaling pathway, which is implicated in CRC progression and resistance. Through comprehensive experiments, they demonstrated the synergistic effects of metformin and ICG-001 in reducing CRC cell viability, inhibiting colony and spheroid formation, and disrupting invasion capabilities, particularly in 5FU-resistant CRC cells and CSCs. This combined treatment induced apoptosis and autophagy while decreasing the expression of CSC markers, offering a potential strategy to target CSCs and overcome chemotherapy resistance in CRC [100].

Xiaoli Zhang et al. addressed drug resistance in CRC by targeting drug-tolerant persister (DTP) cells through GPX4 inhibition. Their study aimed to eliminate resilient CRC cells by inducing ferroptosis, a specific form of cell death. They established models of DTP cells derived from CRC cell lines and uncovered key characteristics of these cells, including reduced drug sensitivity and a state of quiescence. Their findings suggest that targeting GPX4 to induce ferroptosis offers a promising therapeutic strategy to overcome drug resistance in CRC. Experimental data from in vivo models confirmed the efficacy of GPX4 inhibitors in preventing tumor regrowth following anticancer drug treatment cessation [101]. 

Youran Li et al. contributed to our understanding of CRC by investigating the role of long non-coding RNA (lncRNA) LINC01315 in CSCs and exosomal communication. Their research elucidated how LINC01315, overexpressed in CRC stem cells characterized by CD133+/CD44+ markers, contributes to CRC malignancy by enhancing proliferation, migration, and stemness. They demonstrated that LINC01315 plays a crucial role in sustaining the aggressive phenotype of CRC stem cells and can be packaged into exosomes, thereby influencing intercellular communication in the tumor microenvironment. Their findings suggest LINC01315 as a potential biomarker and therapeutic target for CRC, offering new avenues for intervention to curb CRC progression and overcome chemotherapy resistance. Additionally, their bioinformatics analysis identified potential interactions between LINC01315 and the genes involved in cancer progression, providing further insights into therapeutic targets for CRC [102].

Through the application of CRISPR-Cas9 technology, Shimokawa and team revealed that targeting LGR5+ CCSCs for elimination in CRC human organoids results in tumor shrinkage in xenograft models derived from these organoids. Yet, tumor recurrence is observed weeks later, with differentiation of tumor cells back to LGR5+ CCSCs, showcasing the phenomenon of cellular plasticity [103]. Similarly, another study employing CRC organoids with diphtheria toxin receptor expressed under the LGR5 promoter to specifically target LGR5+ CCSCs confirmed these findings [104]. The eradication of CCSCs curtails the initial tumor expansion but does not halt tumor resurgence at the original site after treatment cessation, attributed to the proliferation of LGR5− cells, although it impacts metastatic sites. This highlights the potential of targeted CSC depletion in preventing distant metastases, offering a promising strategy for treating metastatic conditions. The significance of cellular plasticity in metastasis formation and niche repopulation remains underexplored [105]. 

The TME is suggested to amplify this cellular flexibility. Therefore, the complexity of cellular plasticity and the influence of the TME hinder the development of novel treatments and complete cancer eradication, underscoring the insufficient nature of solely targeting CCSCs. The variability and evolving characteristics of CCSCs pose additional targeting challenges. Lenos et al. utilized a marker-independent quantitative approach to study colon cancer growth, revealing that CSC functionality does not exclusively reside in cells marked as CSCs. They found that any tumor cell could drive growth in conducive conditions, especially near the tumor’s periphery where CAFs are present [106]. This suggests that CSC roles in established tumors are defined more by their location and timing, heavily influenced by the TME. Consequently, the adaptability of tumors to the loss of critical components, facilitated by cellular plasticity and the TME, compromises treatment effectiveness [107]. Thus, targeting the TME alongside other resistance mechanisms is vital in the development of new therapeutic approaches as it plays a pivotal role in protecting CSCs from treatment and in supporting primary and metastatic tumor development. Table 2 details the key elements of the TME that significantly influence CSCs, especially focusing on CCSCs, highlighting their critical role.

## 7. Therapeutic Strategies against CCSCs to Overcome Therapy Resistance

In the battle against chemoradiotherapy resistance in cancer, focusing on cancer CSCs offers a groundbreaking pathway. This strategy unfolds through several avenues: driving CSCs towards differentiation, curbing distinct signaling or metabolic routes, leveraging inhibitors to stall cell cycle progression, and integrating miRNA modulation with established therapeutic protocols. A vital component of this strategy involves the exploration and implementation of clinical trials (Table 3) designed to evaluate the efficacy of CSC-targeted therapies.

At the forefront of colorectal CRC diagnostics and treatment are biomarker proteins, which enable precise targeting of therapies. Scientists harness specific antibodies or ligands that latch onto CSC markers, refining the precision of interventions. Among these, MCLA-158 stands out, a dual-action antibody that zeroes in on EGFR and Lgr5. This agent not only curtails the growth of CRC organoids but also exhibits potent antitumor effects in patient-derived models, showing particular efficacy against tumors with enhanced Lgr5 and EGFR levels, including those harboring KRAS mutations that evade cetuximab treatment. Current clinical investigations are broadening MCLA-158’s potential application across a range of solid tumors. Moreover, catumaxomab, celebrated as the pioneering T cell-binding bispecific antibody for treating malignant ascites, showcases its prowess in annihilating CD133+/EpCAM+ CSCs in advanced-stage cancers. This marks significant progress in targeting epithelial cancer CSCs. The realm of therapeutic innovation has expanded to embrace oncolytic virotherapies and CSC-specific vaccines. These strategies utilize viruses engineered to selectively annihilate tumor cells while sparing healthy tissue, sparking targeted immune responses against CSCs. Viruses tailored with a CD133-targeting sequence, for instance, have successfully purged CD133+ CSCs, demonstrating a promising reduction in tumor proliferation in xenograft studies [108,109].

The treatment landscape is further enriched by interventions aimed at key signaling pathways integral to CSC functionality—self-renewal, proliferation, apoptosis, and angiogenesis. Inhibitors like TNIK inhibitors and EGCG are deployed against the Wnt signaling pathway, a crucial element for epithelial stem cell renewal and a perpetrator in colorectal carcinogenesis when aberrant, to mitigate CSC stemness. Similarly, targeting the Hh and Notch pathways, pivotal for tissue development and cellular differentiation, respectively, with specific inhibitors modifies CSC behavior and therapy responsiveness. In the same vein, interventions against the PI3K/Akt/mTOR and JAK/STAT3 pathways, linked to cancer progression and metastasis, are pursued to dampen CSC proliferation and stemness [64,108,109].

Addressing CRC’s hallmark of genomic instability, characterized by chromosomal instability (CIN) and microsatellite instability (MSI), unveils additional therapeutic opportunities. Efforts to counteract Twist1’s upregulation, a driver of CIN that fosters CSC development and resistance, could unlock novel treatment avenues. Enhancing CSCs’ sensitivity to treatments entails reprogramming the TME to bolster immunotherapy efficacy and disrupt CSC–TME interaction. This approach necessitates accounting for the unique cell cycle position of CSCs, typically resistant to conventional therapies, and targeting specific pathways and signals pivotal for CSC transformation and survival. Targeted metabolic reprogramming aims to confront CRC by altering the distinct mitochondrial configurations and glucose metabolism of CSCs. Inhibiting pathways like GLUT1 or the KRAS–JNK axis presents a viable strategy to diminish CSC stemness and resistance, charting a new course for combatting CRC. Emerging therapeutic strategies, including clinical trials investigating the synergistic use of metformin and CSC-targeted dendritic cell vaccines, illuminate the potential to develop cutting-edge immunotherapies [108,109].

The research conducted by Anna Citarella and colleagues delved into the mechanisms behind CRC cells’ resistance to chemotherapy treatments, particularly focusing on cells with KRAS and BRAF mutations. Their study revealed how the HH-GLI and NOTCH signaling pathways play a pivotal role in this resistance. By utilizing a variety of experimental approaches, including cell culture, organoid development, and various molecular assays, Citarella et al. provided insightful evidence into the potential of targeting these pathways to overcome chemotherapeutic resistance. The findings from Citarella and team underscored the significance of the HH-GLI and NOTCH signaling pathways in sustaining CRC cell survival and proliferation despite treatment with 5-fluorouracil (5-FU), a commonly used chemotherapeutic agent. By treating CRC cell lines and organoids with inhibitors targeting these pathways, either alone or in conjunction with 5-FU, the research highlighted a marked decrease in cancer cell viability and invasiveness. This approach notably affected the expression of genes associated with cancer stemness and EMT, a processes integral to cancer metastasis and resistance to treatments. One of the standout observations from Citarella et al.’s work was the efficacy of Arsenic Trioxide (ATO) in simultaneously inhibiting both the HH-GLI and NOTCH pathways. This dual inhibition, especially when combined with 5-FU treatment, significantly curtailed the mesenchymal characteristics of CRC cells, suggesting a promising strategy to bolster the effectiveness of chemotherapy in CRC patients harboring KRAS or BRAF mutations [110].

Recent studies show that napabucasin, a naturally occurring naphthoquinone isolated from plants, exhibits potent anticancer properties and is currently being evaluated in clinical trials. This compound has gained attention for its ability to inhibit cancer stemness by targeting the STAT3 pathway, which plays a crucial role in tumor growth, survival, and the regulation of inflammation within the tumor microenvironment. Additionally, napabucasin acts as a substrate for NQO1, an enzyme involved in the bioactivation of certain drugs, marking it as a bioactivatable drug that leverages NQO1’s mechanism for its anticancer activities. These activities span a wide spectrum, including inhibition of cell proliferation, induction of apoptosis, disruption of cell cycles, suppression of metastasis, and overcoming drug resistance in a diverse range of cancers from myeloid leukemia to hypopharyngeal cancer. One of the standout features of napabucasin is its ability to induce apoptosis through the activation of both intrinsic and extrinsic pathways, significantly elevating the expression of cleaved caspase-3 and PARP in various cancer cell lines and showing promise as a treatment for drug-resistant cancers. Furthermore, napabucasin’s impact on cell cycle regulation provides a new avenue for anticancer drug development, with the ability to arrest the cell cycle at different phases depending on the type of cancer, similar to the action of FDA-approved CDK4/6 inhibitors for breast cancer. Moreover, napabucasin has been effective in reducing metastasis and enhancing drug sensitivity in cancer cells while notably suppressing cancer stemness, a key driver of cancer relapse and metastasis. This suppression is evidenced by the reduced expression of stemness markers and diminished sphere and colony formation abilities, highlighting napabucasin’s potential as a multifaceted anticancer agent. Molecularly, aside from inhibiting STAT3, napabucasin targets NQO1 to generate reactive oxygen species, contributing to its anticancer efficacy. Demonstrated both in vitro and in vivo, napabucasin’s anticancer effects, coupled with ongoing clinical trials showing its synergistic effects with conventional chemotherapy, underscore its potential as a promising anticancer therapy. However, clinical application of napabucasin requires careful monitoring of side effects, particularly gastrointestinal disturbances, to balance its therapeutic benefits with potential risks [111,112].

A study conducted by Zhengguang Li et al. demonstrated the pivotal role of Dishevelled-3 (DVL3) in the progression and prognosis of CRC, presenting a potential therapeutic target for managing CCSCs. Their research highlighted DVL3’s overexpression in CRC tissues and cell lines, correlating significantly with advanced stages of nodal metastasis and poorer patient survival rates [113]. These findings suggest that DVL3 not only contributes to CRC aggressiveness but may also serve as a prognostic indicator. Zhengguang Li et al.’s investigation into the functional role of DVL3 revealed its enhancement of CRC cells’ metastatic potential, including increased migration, invasion, and the promotion of EMT. Through a series of in vitro experiments, the study showed that manipulating the DVL3 levels directly affected EMT marker expression, suggesting that DVL3 drives EMT-like molecular changes in CRC cells. Moreover, the study explored the mechanism behind DVL3’s action, implicating the Wnt/β-catenin signaling pathway in DVL3-mediated effects on CRC stemness and EMT phenotypes. The activation of this pathway by DVL3 was shown to upregulate stemness markers and enhance the mesenchymal phenotype, reinforcing the idea that DVL3 supports CRC progression through regulation of CSC properties and EMT. Importantly, interventions targeting DVL3, such as silencing or pharmacological inhibition, were found to impair the tumorigenic and metastatic capabilities of CRC cells both in vitro and in vivo. These interventions led to reduction in the expression of stemness and EMT markers, decreased migratory and invasive abilities of CRC cells, and heightened sensitivity to chemotherapy, suggesting a potential therapeutic approach to managing CRC by targeting CSCs through the modulation of DVL3 and the Wnt/β-catenin pathway [113].

Recent findings have underscored the pivotal role of ALDH1B1 in CRC, bringing to light its potential as both a biomarker and a therapeutic target. Elevated levels of ALDH1B1 have been consistently observed in human CRC tissues and cell lines, highlighting its integral involvement in the molecular dynamics of colon cancer. This increase in ALDH1B1 expression is notable not only in colorectal adenomas and adenocarcinomas but also in stages of CRC nodal metastasis, indicating a significant role in disease progression. ALDH1B1’s association with stem-like properties in CRC has also been extensively documented. The enzyme’s involvement in key cellular signaling pathways such as Wnt/β-catenin, Notch, and PI3K/Akt suggests its critical function in maintaining CSC characteristics. This linkage positions ALDH1B1 as a potential CSC marker in CRC, further implicating it in cancer aggressiveness and resistance to therapy. High ALDH1B1 expression has been correlated with increased migration, chemoresistance, altered cell cycle regulation, and an enhanced DNA damage response in CRC cells, underlining its multifaceted role in cancer advancement and therapeutic resistance. Given the crucial role of ALDH1B1 in CRC and its association with adverse patient outcomes, targeting this enzyme has emerged as a promising strategy to improve treatment efficacy. The discovery of compounds that inhibit ALDH1B1 activity within cells offers new avenues for enhancing therapy. Inhibition of ALDH1B1 has been shown to reduce colon spheroid and xenograft tumor growth, accompanied by downregulation of CCSC markers. Such findings highlight the therapeutic potential of targeting ALDH1B1 to combat CRC’s stemness and aggressiveness, proposing a novel approach to addressing the complexities of CRC treatment and management [114].

## 8. Conclusions

The dynamic landscape of CRC research is increasingly recognizing the pivotal role of colorectal cancer stem cells in mediating therapy resistance, recurrence, and metastasis. Looking toward the future, the path to improving CRC outcomes hinges on novel strategies targeting these elusive cells. Here, we explore promising directions for research and therapeutic development aimed at eradicating CCSCs and enhancing patient care.

Advancing the molecular characterization of CCSCs is paramount, necessitating the identification of new surface markers and the elucidation of their genetic, epigenetic, and metabolic landscapes. High-throughput technologies, single-cell sequencing, and CRISPR-Cas9 gene editing will be instrumental in uncovering the complexities of CCSCs, presenting new therapeutic targets.

Targeting key signaling pathways such as Wnt/β-catenin, Notch, Hedgehog (Hh), and Hippo, which are aberrantly activated in CCSCs, offers fertile ground for therapeutic interventions. Developing small-molecule inhibitors, monoclonal antibodies, and combination therapies will be crucial to modulate these pathways effectively and overcome resistance mechanisms. The tumor microenvironment (TME) plays a supportive role in CCSC survival and proliferation. Strategies aiming to disrupt this niche, including targeting cancer-associated fibroblasts (CAFs), modulating the immune microenvironment, and inhibiting angiogenesis, are essential for therapeutic efficacy.

CCSCs exhibit unique metabolic profiles that confer survival advantages and therapy resistance. Exploiting these metabolic vulnerabilities represents a novel approach to eliminate CCSCs. Developing drugs that disrupt crucial metabolic pathways for CCSC maintenance and expansion is urgently needed. Innovations in drug delivery systems that overcome the TME barriers and specifically target CCSCs are critical. Nanotechnology-based carriers, antibody–drug conjugates, and engineered oncolytic viruses promise to enhance drug efficacy while minimizing toxicity. Immunotherapy presents a burgeoning field aiming to harness the immune system against CCSCs. Identifying CCSC-specific antigens and developing CAR-T cell therapies, cancer vaccines, and checkpoint inhibitors could revolutionize CRC treatment.

Initiating clinical trials to evaluate CCSC-targeted therapies and integrating molecular profiling into clinical practice will enable personalized medicine approaches in CRC, ensuring that patients receive treatments tailored to their tumor’s molecular characteristics. It is essential to understand and overcome CCSC-mediated drug resistance. Future research should focus on unveiling new molecular targets and developing therapies that prevent or reverse resistance, ensuring the long-term efficacy of CRC treatments.

As we venture into a new era of CRC treatment, focusing on CCSCs offers hope for overcoming therapy resistance and disease recurrence. Integrating advanced molecular insights with innovative therapeutic strategies and personalized medicine will pave the way for breakthroughs in CRC treatment, bringing us closer to the ultimate goal of curing this formidable disease.

## 9. Future Perspectives

A major hurdle in the realm of preclinical research is the translation of findings into tangible clinical benefits for patients suffering from CRC [115]. Unfortunately, a significant number of clinical trials fail to validate the positive outcomes of new drug treatments due to either their ineffectiveness in combating cancer in patients or the occurrence of side effects that halt the continuation of the trial. Future clinical trial designs must take into consideration the heterogeneity present within and across CRC tumors, which plays a crucial role in how patients respond to treatments. The introduction of targeted therapies and immunotherapies in recent years has significantly improved the survival rates of CRC patients, with novel treatments broadening the spectrum of options for patients with advanced CRC that possess particular genetic mutations [9]. However, in spite of the initial success of conventional therapies, most drugs do not effectively target the MRD associated with CSCs, leading to a high relapse rate among patients. Disturbingly, about half of the patients diagnosed with early-stage CRC are likely to develop metastatic disease, and a considerable portion of these cases are deemed non-operable due to the metastases’ size, location, or extent [116].

Future investigations need to focus on designing trials that assess medications that are potentially beneficial for both the initial and later stages of CRC management. The shortfall in exacting preclinical frameworks that encompass both inherent and environmental tumor properties, including subsets of CSCs, the tumor’s structural framework, and the TME, remains a substantial scientific hurdle. The techniques for CCSC isolation and analysis reviewed herein reveal the shortcomings of the existing practices, notably those dependent on CCSC markers. Sorting cells by phenotypic traits only identifies a segment of the CCSC cohort due to their diversity, adaptability, and responsiveness to TME influences. Hence, the implementation of novel methodologies like SdFFF, which categorizes cells based on non-marker traits, or the integration of various isolation strategies, is pivotal. Ultimately, the creation of more refined preclinical models is crucial as the current methods do not adequately determine treatments that could prove to be clinically effective, with a focus on targeting CCSCs [30,117,118].

## Figures and Tables

**Table 1:** CCSC surface markers.

CCSC Marker	Biological Function	Prognostic Significance
**CD24**	Facilitates cell adhesion and serves as a P-selectin ligand	High cytoplasmic CD24 expression is closely tied to decreased patient survival.
**CD29**	Plays a role in cell adhesion processes	Increased expression of CD29 correlates with a poorer prognosis and increased aggressiveness
**CD44**	Regulates cell interactions, adhesion, and migration	Elevated CD44 levels linked to lymph node metastasis, distant metastases, and worse prognosis
**CD44v6**	Binds hepatocyte growth factor, facilitating migration and metastases	High levels of CD44v6 negatively impact overall survival
**CD133**	Governs self-renewal and contributes to tumor angiogenesis	CD133 expression correlates with decreased survival in CRC patients
**CD166**	Mediates homophilic interactions among cells	Increased expression of CD166 associated with shortened patient survival
**EpCAM**	Controls cell adhesion, proliferation, and migration	Loss of EpCAM expression linked to advanced tumor stage, lymph node and distant metastases, and poor prognosis
**Lgr5**	Serves as a downstream target of the Wnt pathway involved in self-renewal	Lgr5 expression linked to lymph node and distant metastases, and overexpression related to reduced overall survival
**Bmi-1**	Functions as a self-renewal regulator	High Bmi-1 expression is associated with poor survival rates
**CD26**	Promotes invasion and metastases	Elevated CD26 expression associated with advanced tumor staging and worse survival

**Table 2:** Based on Ref. [75].

Cells	Impact on Cancer Progression	Influence on CSC Dynamics	Pathways
**Cancer-Associated Fibroblasts**	Facilitates cancer advancement, invasion, and orchestrates morphological transformations through the release of growth-promoting substances such as HGF, CCL12, fibroblast growth factors, and stanniocalcin.	Enhances the characteristics of CSCs and their capacity for invasive metastasis.	Triggers Wnt/β-catenin pathway activation through the release of HGF, matrix metalloproteinases, and cytokines such as TNF-α.
**Adipocytes**	Produces signaling molecules, including leptin, adiponectin, IL-6, MCP-1, and TNF-α.	The leptin receptor sustains an autocatalytic signaling loop that amplifies the CSC population and accelerates tumor expansion.	-
**Tumor-Associated Macrophages**	Fosters tumor growth by triggering T cell inactivity, influencing ECM dynamics, tissue repair, and new blood vessel formation.	Facilitates CSC development through the involvement of Milk-fat globule-EGF factor 8 (MFG-E8).	Initiates STAT3 and Hedgehog signaling, promoting cancer cell growth and resistance to treatment in CSCs.
**T-regulatory cells**	Inhibits immune responses by releasing cytokines such as IL-10, IL-35, and TGF-β, and dampens the activity of cytotoxic T cells and NK cells.	In low-oxygen conditions, FOXP3+ Treg cells produce IL-17, leading to the growth of the CCSC population, as shown by elevated levels of CD133, CD44s, and EpCAM. Additionally, these cells modulate CSC characteristics by emitting prostaglandin (PGE2) via the NF-κB pathway.	IL-17 triggers the activation of the Akt and MAPK signaling pathways
**Myeloid-Derived Suppressor Cells**	They release arginase 1, reactive oxygen species (ROS), and inducible nitric oxide synthase (iNOS) to suppress the tumor-fighting abilities of NK cells and T cells, aiding in immune evasion and facilitating the onset and advancement of tumors.	Their presence in the intestinal mucosa is tied to the activation of CXCR2 on endothelial and immune cells within colorectal cancer regions.	-
**HIF**	HIF-1α and HIF-2α interact with hypoxia response elements (HRE), exacerbating tumor development, infiltration, and spread.	HIF-1α enhances the gene expression driven by β-catenin within the traditional Wnt pathway. Hypoxia’s role in keeping CSCs dormant further aids in their resistance to therapeutic drugs.	Wnt/β-catenin

**Table 3:** Clinical trials revised from www.clinicaltrials.gov (accessed on 2 April 2024).

Trial Number	Clinical Study	Interventions	Aim
**NCT00004087**	Radiolabeled monoclonal antibody therapy plus peripheral stem cell transplantation in treating patients with metastatic or recurrent colorectal cancer or pancreatic cancer	Biological: filgrastim Procedure: autologous bone marrow transplantation Procedure: peripheral blood stem cell transplantation Radiation: indium In 111 monoclonal antibody MN-14 Radiation: yttrium Y 90 monoclonal antibody MN-14	Effectiveness of combined treatment involving radiolabeled monoclonal antibodies and peripheral stem cell transplantation in treating metastatic or recurrent colorectal cancer unresponsive to prior therapies.
**NCT01075893**	Changes in stem cells of the colon in response to increased risk of colorectal cancer	Not provided	Exploration of stem cell frequency and distribution among individuals at high and normal risk for colorectal cancer. Enhanced cell proliferation at the crypt apex in high-risk patients is attributed to alterations in the stem cell count at the crypt base.
**NCT01286883**	Cancer stem cell markers and prognostic markers in circulating tumor cells	Not provided	Analyzing the genetic profiles of circulating and primary tumors to determine the prevalence of cancer cell genotypes in patients exhibiting elevated circulating tumor cell counts or experiencing early disease recurrence.
**NCT01483001**	Feasibility study on stem cells sensitivity assay	Cancer Stem Cells Sensitivity Assay To test in vitro sensitivity of cancer stem cells to several antineoplastic drugs in order to personalize treatment	Isolation and identification of cancer stem cells within solid tumors, including colorectal cancer.
**NCT01577511**	Invasiveness and chemoresistance of cancer stem cells in colon cancer	Biological: Samples and followup	Exploration of the traits related to the spread and drug resistance of colorectal cancer stem cells, along with their genetic characteristics.
**NCT02176746**	A phase I/II study of active immunotherapy with cancer stem cells vaccine for CRC	Biological: cancer stem cell vaccine	The investigation evaluated the anticancer immune responses elicited by cytotoxic T-cells and B-cell antibodies, both activated through exposure to dendritic cells derived from colorectal cancer stem cells.
**NCT03002727**	Role of CD133 and microsatellite status in evaluation of rectosigmoid cancer; young adults received neoadjuvant treatment	Not provided	The study aims to explore the potential link between microsatellite status and the prevalence of colorectal cancer stem cells, examining how this relationship could influence the outcomes of the disease and the effectiveness of treatment strategies.
**NCT03803241**	CD133+ cell infusion in patients with colorectal liver metastases	Drug: CD133+ infusion Other: portal vein embolization	Patients deemed unsuitable for surgery underwent treatment with CD133+ cell infusion and portal vein embolization to potentially qualify them for surgical intervention.
**NCT02753127**	A Study of Napabucasin (BBI-608) in Combination With FOLFIRI in Adult Patients With Previously Treated Metastatic Colorectal Cancer (CanStem303C)	Drug: Napabucasin	To evaluate the impact of combining napabucasin with biweekly FOLFIRI, compared to biweekly FOLFIRI alone, with or without the addition of bevacizumab, on the overall survival of individuals with metastatic colorectal cancer who have undergone previous treatments.

## Data Availability

Data sharing is not applicable to this article.

## References

[1] Siegel R.L., Miller K.D., Goding Sauer A., Fedewa S.A., Butterly L.F., Anderson J.C., Cercek A., Smith R.A., Jemal A. (2020). Colorectal cancer statistics, 2020. CA Cancer J. Clin..

[2] Cancer Genome Atlas N. (2012). Comprehensive molecular characterization of human colon and rectal cancer. Nature.

[3] Zhang B., Wang J., Wang X., Zhu J., Liu Q., Shi Z., Chambers M.C., Zimmerman L.J., Shaddox K.F., Kim S. (2014). Proteogenomic characterization of human colon and rectal cancer. Nature.

[4] Inamura K. (2018). Colorectal Cancers: An Update on Their Molecular Pathology. Cancers.

[5] Punt C.J., Koopman M., Vermeulen L. (2017). From tumour heterogeneity to advances in precision treatment of colorectal cancer. Nat. Rev. Clin. Oncol..

[6] Tran E., Robbins P.F., Lu Y.C., Prickett T.D., Gartner J.J., Jia L., Pasetto A., Zheng Z., Ray S., Groh E.M. (2016). T-Cell Transfer Therapy Targeting Mutant KRAS in Cancer. N. Engl. J. Med..

[7] Overman M.J., McDermott R., Leach J.L., Lonardi S., Lenz H.J., Morse M.A., Desai J., Hill A., Axelson M., Moss R.A. (2017). Nivolumab in patients with metastatic DNA mismatch repair-deficient or microsatellite instability-high colorectal cancer (CheckMate 142): An open-label, multicentre, phase 2 study. Lancet Oncol..

[8] Miyamoto Y., Suyama K., Baba H. (2017). Recent Advances in Targeting the EGFR Signaling Pathway for the Treatment of Metastatic Colorectal Cancer. Int. J. Mol. Sci..

[9] Hervieu C., Christou N., Battu S., Mathonnet M. (2021). The Role of Cancer Stem Cells in Colorectal Cancer: From the Basics to Novel Clinical Trials. Cancers.

[10] Lea D., Haland S., Hagland H.R., Soreide K. (2014). Accuracy of TNM staging in colorectal cancer: A review of current culprits, the modern role of morphology and stepping-stones for improvements in the molecular era. Scand. J. Gastroenterol..

[11] Printz C. (2010). New AJCC cancer staging manual reflects changes in cancer knowledge. Cancer.

[12] Amin M.B., Greene F.L., Edge S.B., Compton C.C., Gershenwald J.E., Brookland R.K., Meyer L., Gress D.M., Byrd D.R., Winchester D.P. (2017). The Eighth Edition AJCC Cancer Staging Manual: Continuing to build a bridge from a population-based to a more “personalized” approach to cancer staging. CA Cancer J. Clin..

[13] Ciombor K.K., Wu C., Goldberg R.M. (2015). Recent therapeutic advances in the treatment of colorectal cancer. Annu. Rev. Med..

[14] Shinji S., Yamada T., Matsuda A., Sonoda H., Ohta R., Iwai T., Takeda K., Yonaga K., Masuda Y., Yoshida H. (2022). Recent Advances in the Treatment of Colorectal Cancer: A Review. J. Nippon Med. Sch..

[15] Marincas M., Cirimbei C., Prunoiu V., Eliescu A.L., Buzatu R., Stefan I., Bratucu E., Murarasu D., Puiu L., Mihalcea C. (2011). Postradiotherapy regression—A prognostic factor in rectal neoplasm. Chirurgia.

[16] Douaiher J., Ravipati A., Grams B., Chowdhury S., Alatise O., Are C. (2017). Colorectal cancer-global burden, trends, and geographical variations. J. Surg. Oncol..

[17] Sawicki T., Ruszkowska M., Danielewicz A., Niedzwiedzka E., Arlukowicz T., Przybylowicz K.E. (2021). A Review of Colorectal Cancer in Terms of Epidemiology, Risk Factors, Development, Symptoms and Diagnosis. Cancers.

[18] Baidoun F., Elshiwy K., Elkeraie Y., Merjaneh Z., Khoudari G., Sarmini M.T., Gad M., Al-Husseini M., Saad A. (2021). Colorectal Cancer Epidemiology: Recent Trends and Impact on Outcomes. Curr. Drug Targets.

[19] Wang M.Y., Qiu Y.H., Cai M.L., Zhang C.H., Wang X.W., Liu H., Chen Y., Zhao W.L., Liu J.B., Shao R.G. (2020). Role and molecular mechanism of stem cells in colorectal cancer initiation. J. Drug Target..

[20] Munro M.J., Wickremesekera S.K., Peng L., Tan S.T., Itinteang T. (2018). Cancer stem cells in colorectal cancer: A review. J. Clin. Pathol..

[21] Relation T., Dominici M., Horwitz E.M. (2017). Concise Review: An (Im)Penetrable Shield: How the Tumor Microenvironment Protects Cancer Stem Cells. Stem Cells.

[22] Kaushik V., Kulkarni Y., Felix K., Azad N., Iyer A.K.V., Yakisich J.S. (2021). Alternative models of cancer stem cells: The stemness phenotype model, 10 years later. World J. Stem Cells.

[23] Lapidot T., Sirard C., Vormoor J., Murdoch B., Hoang T., Caceres-Cortes J., Minden M., Paterson B., Caligiuri M.A., Dick J.E. (1994). A cell initiating human acute myeloid leukaemia after transplantation into SCID mice. Nature.

[24] Hayat H., Hayat H., Dwan B.F., Gudi M., Bishop J.O., Wang P. (2021). A Concise Review: The Role of Stem Cells in Cancer Progression and Therapy. OncoTargets Ther..

[25] Erkisa M., Karakas D., Ulukaya E. (2019). Cancer Stem Cells: Root of the Evil. Crit. Rev. Oncog..

[26] Gurel C., Inetas G., Hortu I., Tunc E., Kuscu G.C., Dindaroglu F.C., Sahin O., Buhur A., Oktem G. (2019). Cancer and Cancer Stem Cells: New Molecular Perspectives. Crit. Rev. Oncog..

[27] Atashzar M.R., Baharlou R., Karami J., Abdollahi H., Rezaei R., Pourramezan F., Zoljalali Moghaddam S.H. (2020). Cancer stem cells: A review from origin to therapeutic implications. J. Cell. Physiol..

[28] Fedyanin M., Anna P., Elizaveta P., Sergei T. (2017). Role of Stem Cells in Colorectal Cancer Progression and Prognostic and Predictive Characteristics of Stem Cell Markers in Colorectal Cancer. Curr. Stem Cell Res. Ther..

[29] Zhou Y., Xia L., Wang H., Oyang L., Su M., Liu Q., Lin J., Tan S., Tian Y., Liao Q. (2018). Cancer stem cells in progression of colorectal cancer. Oncotarget.

[30] Jahanafrooz Z., Mosafer J., Akbari M., Hashemzaei M., Mokhtarzadeh A., Baradaran B. (2020). Colon cancer therapy by focusing on colon cancer stem cells and their tumor microenvironment. J. Cell. Physiol..

[31] Najafi M., Farhood B., Mortezaee K. (2019). Cancer stem cells (CSCs) in cancer progression and therapy. J. Cell. Physiol..

[32] Desbats M.A., Giacomini I., Prayer-Galetti T., Montopoli M. (2020). Metabolic Plasticity in Chemotherapy Resistance. Front. Oncol..

[33] Batlle E., Clevers H. (2017). Cancer stem cells revisited. Nat. Med..

[34] Melin C., Perraud A., Akil H., Jauberteau M.O., Cardot P., Mathonnet M., Battu S. (2012). Cancer stem cell sorting from colorectal cancer cell lines by sedimentation field flow fractionation. Anal. Chem..

[35] De Angelis M.L., Francescangeli F., Zeuner A., Baiocchi M. (2021). Colorectal Cancer Stem Cells: An Overview of Evolving Methods and Concepts. Cancers.

[36] Conciatori F., Bazzichetto C., Falcone I., Ferretti G., Cognetti F., Milella M., Ciuffreda L. (2019). Colorectal cancer stem cells properties and features: Evidence of interleukin-8 involvement. Cancer Drug Resist..

[37] Parizadeh S.M., Jafarzadeh-Esfehani R., Hassanian S.M., Parizadeh S.M.R., Vojdani S., Ghandehari M., Ghazaghi A., Khazaei M., Shahidsales S., Rezayi M. (2019). Targeting cancer stem cells as therapeutic approach in the treatment of colorectal cancer. Int. J. Biochem. Cell Biol..

[38] Zalewski A., Snook A.E., Waldman S.A. (2021). Stem cells as therapeutic targets in colorectal cancer. Pers. Med..

[39] Zeuner A., Todaro M., Stassi G., De Maria R. (2014). Colorectal cancer stem cells: From the crypt to the clinic. Cell Stem Cell.

[40] Akbarzadeh M., Maroufi N.F., Tazehkand A.P., Akbarzadeh M., Bastani S., Safdari R., Farzane A., Fattahi A., Nejabati H.R., Nouri M. (2019). Current approaches in identification and isolation of cancer stem cells. J. Cell. Physiol..

[41] Kondo J., Endo H., Okuyama H., Ishikawa O., Iishi H., Tsujii M., Ohue M., Inoue M. (2011). Retaining cell-cell contact enables preparation and culture of spheroids composed of pure primary cancer cells from colorectal cancer. Proc. Natl. Acad. Sci. USA.

[42] Ricci-Vitiani L., Lombardi D.G., Pilozzi E., Biffoni M., Todaro M., Peschle C., De Maria R. (2007). Identification and expansion of human colon-cancer-initiating cells. Nature.

[43] Vermeulen L., Todaro M., de Sousa Mello F., Sprick M.R., Kemper K., Perez Alea M., Richel D.J., Stassi G., Medema J.P. (2008). Single-cell cloning of colon cancer stem cells reveals a multi-lineage differentiation capacity. Proc. Natl. Acad. Sci. USA.

[44] De Angelis M.L., Zeuner A., Policicchio E., Russo G., Bruselles A., Signore M., Vitale S., De Luca G., Pilozzi E., Boe A. (2016). Cancer Stem Cell-Based Models of Colorectal Cancer Reveal Molecular Determinants of Therapy Resistance. Stem Cells Transl. Med..

[45] Kondo J., Ekawa T., Endo H., Yamazaki K., Tanaka N., Kukita Y., Okuyama H., Okami J., Imamura F., Ohue M. (2019). High-throughput screening in colorectal cancer tissue-originated spheroids. Cancer Sci..

[46] Sato T., Vries R.G., Snippert H.J., van de Wetering M., Barker N., Stange D.E., van Es J.H., Abo A., Kujala P., Peters P.J. (2009). Single Lgr5 stem cells build crypt-villus structures in vitro without a mesenchymal niche. Nature.

[47] Yao Y., Xu X., Yang L., Zhu J., Wan J., Shen L., Xia F., Fu G., Deng Y., Pan M. (2020). Patient-Derived Organoids Predict Chemoradiation Responses of Locally Advanced Rectal Cancer. Cell Stem Cell.

[48] Narasimhan V., Wright J.A., Churchill M., Wang T., Rosati R., Lannagan T.R.M., Vrbanac L., Richardson A.B., Kobayashi H., Price T. (2020). Medium-throughput Drug Screening of Patient-derived Organoids from Colorectal Peritoneal Metastases to Direct Personalized Therapy. Clin. Cancer Res..

[49] Drost J., van Jaarsveld R.H., Ponsioen B., Zimberlin C., van Boxtel R., Buijs A., Sachs N., Overmeer R.M., Offerhaus G.J., Begthel H. (2015). Sequential cancer mutations in cultured human intestinal stem cells. Nature.

[50] Fujii M., Shimokawa M., Date S., Takano A., Matano M., Nanki K., Ohta Y., Toshimitsu K., Nakazato Y., Kawasaki K. (2016). A Colorectal Tumor Organoid Library Demonstrates Progressive Loss of Niche Factor Requirements during Tumorigenesis. Cell Stem Cell.

[51] Fumagalli A., Drost J., Suijkerbuijk S.J., van Boxtel R., de Ligt J., Offerhaus G.J., Begthel H., Beerling E., Tan E.H., Sansom O.J. (2017). Genetic dissection of colorectal cancer progression by orthotopic transplantation of engineered cancer organoids. Proc. Natl. Acad. Sci. USA.

[52] O’Rourke K.P., Loizou E., Livshits G., Schatoff E.M., Baslan T., Manchado E., Simon J., Romesser P.B., Leach B., Han T. (2017). Transplantation of engineered organoids enables rapid generation of metastatic mouse models of colorectal cancer. Nat. Biotechnol..

[53] Sakai E., Nakayama M., Oshima H., Kouyama Y., Niida A., Fujii S., Ochiai A., Nakayama K.I., Mimori K., Suzuki Y. (2018). Combined Mutation of Apc, Kras, and Tgfbr2 Effectively Drives Metastasis of Intestinal Cancer. Cancer Res..

[54] Melin C., Perraud A., Bounaix Morand du Puch C., Loum E., Giraud S., Cardot P., Jauberteau M.O., Lautrette C., Battu S., Mathonnet M. (2014). Sedimentation field flow fractionation monitoring of in vitro enrichment in cancer stem cells by specific serum-free culture medium. J. Chromatogr. B Anal. Technol. Biomed. Life Sci..

[55] Melin C., Perraud A., Christou N., Bibes R., Cardot P., Jauberteau M.O., Battu S., Mathonnet M. (2015). New ex-ovo colorectal-cancer models from different SdFFF-sorted tumor-initiating cells. Anal. Bioanal. Chem..

[56] Matsui W.H. (2016). Cancer stem cell signaling pathways. Medicine.

[57] Zhu Y., Li X. (2023). Advances of Wnt Signalling Pathway in Colorectal Cancer. Cells.

[58] Miyako S., Matsuda T., Koma Y.I., Koide T., Sawada R., Hasegawa H., Yamashita K., Harada H., Urakawa N., Goto H. (2023). Significance of Wnt/beta-Catenin Signal Activation for Resistance to Neoadjuvant Chemoradiotherapy in Rectal Cancer. Biomedicines.

[59] Chen Y., Chen M., Deng K. (2023). Blocking the Wnt/beta-catenin signaling pathway to treat colorectal cancer: Strategies to improve current therapies (Review). Int. J. Oncol..

[60] He K., Gan W.J. (2023). Wnt/β-Catenin Signaling Pathway in the Development and Progression of Colorectal Cancer. Cancer Manag. Res..

[61] Zhao H., Ming T., Tang S., Ren S., Yang H., Liu M., Tao Q., Xu H. (2022). Wnt signaling in colorectal cancer: Pathogenic role and therapeutic target. Mol. Cancer.

[62] Zhu G.X., Gao D., Shao Z.Z., Chen L., Ding W.J., Yu Q.F. (2021). Wnt/beta-catenin signaling: Causes and treatment targets of drug resistance in colorectal cancer (Review). Mol. Med. Rep..

[63] Brisset M., Mehlen P., Meurette O., Hollande F. (2023). Notch receptor/ligand diversity: Contribution to colorectal cancer stem cell heterogeneity. Front. Cell Dev. Biol..

[64] Ebrahimi N., Afshinpour M., Fakhr S.S., Kalkhoran P.G., Shadman-Manesh V., Adelian S., Beiranvand S., Rezaei-Tazangi F., Khorram R., Hamblin M.R. (2023). Cancer stem cells in colorectal cancer: Signaling pathways involved in stemness and therapy resistance. Crit. Rev. Oncol. Hematol..

[65] Zhou B., Lin W., Long Y., Yang Y., Zhang H., Wu K., Chu Q. (2022). Notch signaling pathway: Architecture, disease, and therapeutics. Signal Transduct. Target. Ther..

[66] Das P.K., Islam F., Lam A.K. (2020). The Roles of Cancer Stem Cells and Therapy Resistance in Colorectal Carcinoma. Cells.

[67] Tyagi A., Sharma A.K., Damodaran C. (2020). A Review on Notch Signaling and Colorectal Cancer. Cells.

[68] Lin A., Yao J., Cheng Q., Liu Z., Luo P., Zhang J. (2023). Mutations Status of NOTCH Signaling Pathway Predict Prognosis of Immune Checkpoint Inhibitors in Colorectal Cancer. J. Inflamm. Res..

[69] Negri F., Bottarelli L., Pedrazzi G., Maddalo M., Leo L., Milanese G., Sala R., Lecchini M., Campanini N., Bozzetti C. (2023). Notch-Jagged1 signaling and response to bevacizumab therapy in advanced colorectal cancer: A glance to radiomics or back to physiopathology?. Front. Oncol..

[70] Yahyanejad S., Theys J., Vooijs M. (2016). Targeting Notch to overcome radiation resistance. Oncotarget.

[71] Yuan X., Wu H., Xu H., Xiong H., Chu Q., Yu S., Wu G.S., Wu K. (2015). Notch signaling: An emerging therapeutic target for cancer treatment. Cancer Lett..

[72] Geyer N., Gerling M. (2021). Hedgehog Signaling in Colorectal Cancer: All in the Stroma?. Int. J. Mol. Sci..

[73] Sigafoos A.N., Paradise B.D., Fernandez-Zapico M.E. (2021). Hedgehog/GLI Signaling Pathway: Transduction, Regulation, and Implications for Disease. Cancers.

[74] Usui T., Sakurai M., Umata K., Elbadawy M., Ohama T., Yamawaki H., Hazama S., Takenouchi H., Nakajima M., Tsunedomi R. (2018). Hedgehog Signals Mediate Anti-Cancer Drug Resistance in Three-Dimensional Primary Colorectal Cancer Organoid Culture. Int. J. Mol. Sci..

[75] Gupta R., Bhatt L.K., Johnston T.P., Prabhavalkar K.S. (2019). Colon cancer stem cells: Potential target for the treatment of colorectal cancer. Cancer Biol. Ther..

[76] Giammona A., Crivaro E., Stecca B. (2023). Emerging Roles of Hedgehog Signaling in Cancer Immunity. Int. J. Mol. Sci..

[77] Tiwari A., Saraf S., Verma A., Panda P.K., Jain S.K. (2018). Novel targeting approaches and signaling pathways of colorectal cancer: An insight. World J. Gastroenterol..

[78] Clara J.A., Monge C., Yang Y., Takebe N. (2020). Targeting signalling pathways and the immune microenvironment of cancer stem cells—A clinical update. Nat. Rev. Clin. Oncol..

[79] Piccolo S., Dupont S., Cordenonsi M. (2014). The biology of YAP/TAZ: Hippo signaling and beyond. Physiol. Rev..

[80] Mo J.S., Park H.W., Guan K.L. (2014). The Hippo signaling pathway in stem cell biology and cancer. EMBO Rep..

[81] Park J.H., Shin J.E., Park H.W. (2018). The Role of Hippo Pathway in Cancer Stem Cell Biology. Mol. Cells.

[82] Maugeri-Sacca M., De Maria R. (2018). The Hippo pathway in normal development and cancer. Pharmacol. Ther..

[83] Mohajan S., Jaiswal P.K., Vatanmakarian M., Yousefi H., Sankaralingam S., Alahari S.K., Koul S., Koul H.K. (2021). Hippo pathway: Regulation, deregulation and potential therapeutic targets in cancer. Cancer Lett..

[84] Zinatizadeh M.R., Miri S.R., Zarandi P.K., Chalbatani G.M., Raposo C., Mirzaei H.R., Akbari M.E., Mahmoodzadeh H. (2021). The Hippo Tumor Suppressor Pathway (YAP/TAZ/TEAD/MST/LATS) and EGFR-RAS-RAF-MEK in cancer metastasis. Genes Dis..

[85] Zeng R., Dong J. (2021). The Hippo Signaling Pathway in Drug Resistance in Cancer. Cancers.

[86] Zafari N., Khosravi F., Rezaee Z., Esfandyari S., Bahiraei M., Bahramy A., Ferns G.A., Avan A. (2022). The role of the tumor microenvironment in colorectal cancer and the potential therapeutic approaches. J. Clin. Lab. Anal..

[87] Wozniakova M., Skarda J., Raska M. (2022). The Role of Tumor Microenvironment and Immune Response in Colorectal Cancer Development and Prognosis. Pathol. Oncol. Res..

[88] Vermeulen L., De Sousa E.M.F., van der Heijden M., Cameron K., de Jong J.H., Borovski T., Tuynman J.B., Todaro M., Merz C., Rodermond H. (2010). Wnt activity defines colon cancer stem cells and is regulated by the microenvironment. Nat. Cell Biol..

[89] Novoa Diaz M.B., Martin M.J., Gentili C. (2022). Tumor microenvironment involvement in colorectal cancer progression via Wnt/beta-catenin pathway: Providing understanding of the complex mechanisms of chemoresistance. World J. Gastroenterol..

[90] Nallasamy P., Nimmakayala R.K., Parte S., Are A.C., Batra S.K., Ponnusamy M.P. (2022). Tumor microenvironment enriches the stemness features: The architectural event of therapy resistance and metastasis. Mol. Cancer.

[91] Yang Y., Wang Y. (2021). Role of Epigenetic Regulation in Plasticity of Tumor Immune Microenvironment. Front. Immunol..

[92] Li J., Chen D., Shen M. (2022). Tumor Microenvironment Shapes Colorectal Cancer Progression, Metastasis, and Treatment Responses. Front. Med..

[93] Bregenzer M., Horst E., Mehta P., Snyder C., Repetto T., Mehta G. (2022). The Role of the Tumor Microenvironment in CSC Enrichment and Chemoresistance: 3D Co-culture Methods. Methods Mol. Biol..

[94] Taeb S., Ashrafizadeh M., Zarrabi A., Rezapoor S., Musa A.E., Farhood B., Najafi M. (2022). Role of Tumor Microenvironment in Cancer Stem Cells Resistance to Radiotherapy. Curr. Cancer Drug Targets.

[95] Rainho M.A., Siqueira P.B., de Amorim I.S.S., Mencalha A.L., Thole A.A. (2023). Mitochondria in colorectal cancer stem cells—A target in drug resistance. Cancer Drug Resist..

[96] Wei Z., Wang Z., Chai Q., Li Z., Zhang M., Zhang Y., Zhang L., Tang Q., Zhu H., Sui H. (2023). Exosomes derived from MDR cells induce cetuximab resistance in CRC via PI3K/AKT signaling-mediated Sox2 and PD-L1 expression. Exp. Ther. Med..

[97] Li Y., Yuan Y., Yang L., Chen H., Zhang X., Wen T., Liao W., Zhao M., Zhao Z., Hu Q. (2023). AT7867 Inhibits the Growth of Colorectal Cancer Stem-Like Cells and Stemness by Regulating the Stem Cell Maintenance Factor Ascl2 and Akt Signaling. Stem Cells Int..

[98] Su Y., Choi H.S., Choi J.H., Kim H.S., Jang Y.S., Seo J.W. (2023). 7S,15R-Dihydroxy-16S,17S-epoxy-docosapentaenoic Acid Overcomes Chemoresistance of 5-Fluorouracil by Suppressing the Infiltration of Tumor-Associated Macrophages and Inhibiting the Activation of Cancer Stem Cells in a Colorectal Cancer Xenograft Model. Mar. Drugs.

[99] Mangiapane L.R., Nicotra A., Turdo A., Gaggianesi M., Bianca P., Di Franco S., Sardina D.S., Veschi V., Signore M., Beyes S. (2022). PI3K-driven HER2 expression is a potential therapeutic target in colorectal cancer stem cells. Gut.

[100] Roy S., Zhao Y., Yuan Y.C., Goel A. (2022). Metformin and ICG-001 Act Synergistically to Abrogate Cancer Stem Cells-Mediated Chemoresistance in Colorectal Cancer by Promoting Apoptosis and Autophagy. Cancers.

[101] Zhang X., MaY Y., Ma J., Yang L., Song Q., Wang H., Lv G. (2022). Glutathione Peroxidase 4 as a Therapeutic Target for Anti-Colorectal Cancer Drug-Tolerant Persister Cells. Front. Oncol..

[102] Li Y., Wu M., Xu S., Huang H., Yan L., Gu Y. (2022). Colorectal cancer stem cell-derived exosomal long intergenic noncoding RNA 01315 (LINC01315) promotes proliferation, migration, and stemness of colorectal cancer cells. Bioengineered.

[103] Shimokawa M., Ohta Y., Nishikori S., Matano M., Takano A., Fujii M., Date S., Sugimoto S., Kanai T., Sato T. (2017). Visualization and targeting of LGR5^+^ human colon cancer stem cells. Nature.

[104] de Sousa e Melo F., Kurtova A.V., Harnoss J.M., Kljavin N., Hoeck J.D., Hung J., Anderson J.E., Storm E.E., Modrusan Z., Koeppen H. (2017). A distinct role for Lgr5^+^ stem cells in primary and metastatic colon cancer. Nature.

[105] Fumagalli A., Oost K.C., Kester L., Morgner J., Bornes L., Bruens L., Spaargaren L., Azkanaz M., Schelfhorst T., Beerling E. (2020). Plasticity of Lgr5-Negative Cancer Cells Drives Metastasis in Colorectal Cancer. Cell Stem Cell.

[106] Lenos K.J.M., Miedema D.M., Lodestijn S.C., Nijman L.E., van den Bosch T., Romero Ros X., Lourenço F.C., Lecca M.C., van der Heijden M., van Neerven S.M. (2018). Stem Cell Functionality Is Microenvironmentally Defined during Tumour Expansion and Therapy Response in Colon Cancer. Nat. Cell Biol..

[107] de Sousa E.M.F., de Sauvage F.J. (2019). Cellular Plasticity in Intestinal Homeostasis and Disease. Cell Stem Cell.

[108] Damane B.P., Marima R., Mulaudzi T.V., Dlamini Z. (2023). Targeting Stem Cells in the Colorectal Cancer Microenvironment to Avert Drug Resistance in Pursuit of Novel Oncotherapies. J. Biol. Regul. Homeost. Agents.

[109] Zhao H., Han R., Wang Z., Xian J., Bai X. (2023). Colorectal Cancer Stem Cells and Targeted Agents. Pharmaceutics.

[110] Citarella A., Catanzaro G., Besharat Z.M., Trocchianesi S., Barbagallo F., Gosti G., Leonetti M., Di Fiore A., Coppola L., Autilio T.M. (2023). Hedgehog-GLI and Notch Pathways Sustain Chemoresistance and Invasiveness in Colorectal Cancer and Their Inhibition Restores Chemotherapy Efficacy. Cancers.

[111] Shao Z., Wang H., Ren H., Sun Y., Chen X. (2023). The Anticancer Effect of Napabucasin (BBI608), a Natural Naphthoquinone. Molecules.

[112] Jing B., Guo F., An R., Gao Y., Li Y., Xie Y., Wang J., Chen Y., Li H., Gao T. (2023). Apoptotic tumor cell-derived microparticles loading Napabucasin inhibit CSCs and synergistic immune therapy. J. Nanobiotechnol..

[113] Li Z., Yang Z., Liu W., Zhu W., Yin L., Han Z., Xian Y., Wen J., Tang H., Lin X. (2023). Disheveled3 enhanced EMT and cancer stem-like cells properties via Wnt/beta-catenin/c-Myc/SOX2 pathway in colorectal cancer. J. Transl. Med..

[114] Tsochantaridis I., Roupas A., Mohlin S., Pappa A., Voulgaridou G.P. (2023). The Concept of Cancer Stem Cells: Elaborating on ALDH1B1 as an Emerging Marker of Cancer Progression. Life.

[115] Sonbol M.B., Ahn D.H., Bekaii-Saab T. (2019). Therapeutic Targeting Strategies of Cancer Stem Cells in Gastrointestinal Malignancies. Biomedicines.

[116] Atreya C.E., Yaeger R., Chu E. (2017). Systemic Therapy for Metastatic Colorectal Cancer: From Current Standards to Future Molecular Targeted Approaches. Am. Soc. Clin. Oncol. Educ. Book..

[117] Takebe N., Miele L., Harris P.J., Jeong W., Bando H., Kahn M., Yang S.X., Ivy S.P. (2015). Targeting Notch, Hedgehog, and Wnt pathways in cancer stem cells: Clinical update. Nat. Rev. Clin. Oncol..

[118] Ogunwobi O.O., Mahmood F., Akingboye A. (2020). Biomarkers in Colorectal Cancer: Current Research and Future Prospects. Int. J. Mol. Sci..

